# Silicon-iron modified biochar remediates cadmium and arsenic co-contaminated paddy soil by regulating cadmium and arsenic speciation

**DOI:** 10.3389/fmicb.2025.1579213

**Published:** 2025-04-11

**Authors:** Yao Chen, Xin Tian, Jia-hao Wang, Yu Zhang, Jie Wang, Zhang-tao Li, Ke-li Zhao, Ji-zi Wu

**Affiliations:** ^1^College of Environment and Resources, Zhejiang A&F University, Lin'an, China; ^2^Key Laboratory of Soil Remediation and Quality Improvement in Zhejiang Province, Zhejiang A&F University, Lin'an, China; ^3^Key Laboratory of Recycling and Eco-Treatment of Waste Biomass of Zhejiang Province, School of Environment and Natural Resources, Zhejiang University of Science and Technology, Hangzhou, China

**Keywords:** modified biochar, cadmium, arsenic, heavy metal speciation, functional genes

## Abstract

**Introduction:**

Silicon–iron-modified biochars (SMBCs) were produced to remediate paddy soil contaminated with both cadmium (Cd) and arsenic (As). This study explored the effects of SMBCs on the transformation of Cd and As species in soil and the associated responses of functional genes to elucidate the remediation mechanisms.

**Method:**

Three silicon–iron modified biochars were utilized. (i) Silicon dioxide magnetic biochar (SMBC1), (ii) Calcium silicate magnetic biochar (SMBC2), and (iii) Sodium silicate magnetic biochar (SMBC3) were applied to paddy soil.

**Results and discussion:**

SMBCs increased the soil pH and the concentration of dissolved organic carbon (DOC) by 0.42–0.54 units and 6.6–16.39%, respectively. SMBC treatments reduced the bioavailable concentrations of Cd and As by 29.09–73.63% and 1.67–8.37%, respectively, transforming As(III) into less toxic As(V) and stabilizing soluble Cd into a more inert residual form. Compared to the control, SMBC significantly increased residual Cd concentrations by 2.94–16.17% (*p* < 0.05) and As(V) concentrations by 11.42–26.07% (*p* < 0.05). Adding calcium silicate (CaSiO3) at a mass ratio of 5% to magnetic biochar resulted in a residual Cd concentration of 0.79 mg·kg^−1^ (an increase of 16.86%) and an As(V) concentration of 37.89 mg·kg^−1^. SMBCs enhanced soil porosity, microbial aioA genes, and sulfate-reducing bacteria, facilitating the oxidation of As(III). Magnetic biochar amended with 5% (CaSiO_3_) (SMBC2) demonstrated superior efficacy in addressing the co-contamination of Cd and As. The remediation mechanisms include the following: (i) an increase in soil pH and a decrease in dissolved organic carbon (DOC), (ii) enhanced *aioA* gene activity, promoting the oxidation of As(III) to As(V), and increased *dissimilatory sulfite reductase beta subunit (DsrB)* gene activity, facilitating the reduction of sulfate ion (SO_4_^2−^) to sulfide ion (S^2−^), leading to the formation of cadmium sulfide (CdS) precipitates and additional precipitation involving As and Fe. These results highlight the potential of calcium silicate–modified magnetic biochar as an effective additive for Cd and As co-contaminated soils, providing insights into heavy metals’ stabilization and transformation mechanisms.

## Graphical abstract

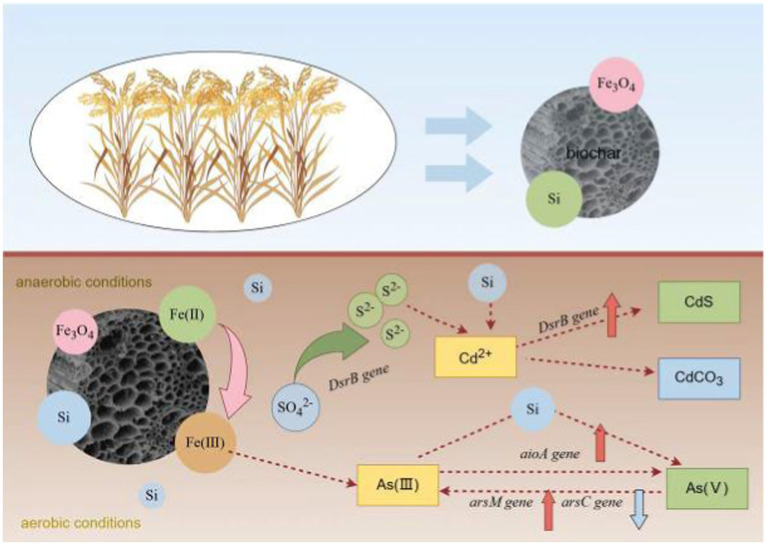

## 1 Introduction

Arsenic (As) and cadmium (Cd) are highly toxic heavy metals. According to the 2014 China Soil Pollution Survey Bulletin, Cd and As rank as the first and third most prevalent inorganic pollutants in Chinese soils, with exceedance rates of 7.0 and 2.7%, respectively. Globally, the Agency for Toxic Substances and Disease Registry classifies them as the seventh and first most hazardous substances, respectively (Yang et al., 2023). Cd and As pose serious environmental risks and can exist in the form of co-pollution, which enters the human body through the food chain and harms human health (Zhao et al., 2012). The co-contamination of Cd and As in paddy soils has become a significant environmental problem in China, posing a serious threat to food security (Panseriya et al., 2020), as rice is an extremely important crop for Chinese people. The contrasting geochemical behaviors of Cd and As make it challenging to simultaneously reduce their mobility in paddy soil (Zwolak, 2020). The bioavailability of Cd and As responds oppositely to changes in soil Eh and pH. For example, Cd forms insoluble precipitates with sulfur, while As is adsorbed onto iron oxides released into the soil (Yang et al., 2021). Consequently, there is an urgent need to control the migration and transformation dynamics of Cd and As in paddy soils to reduce their accumulation in rice and the environmental risk in paddy soil. Biochar is a commonly used soil additive for cation heavy metals. However, due to electrostatic repulsion and elevated soil pH levels, the addition of biochar usually increases the mobility of As. Iron oxides provide an effective solution for adsorbing As through complexation (Pan et al., 2010). Thus, magnetic biochar, produced by loading magnetic materials onto biochar, can effectively remediate both cationic and anionic heavy metals. However, our previous study found that the addition of magnetic biochar reduced As accumulation in rice grains but elevated Cd concentration in those grains, while the CaCl_2_-extracted Cd concentration decreased. Additionally, high doses of magnetic biochar can cause iron toxicity, inhibiting rice growth.

Silicon is indispensable in various aspects, such as the growth and development of rice, nutrient absorption, yield and quality formation, and stress physiology. It can also reduce the accumulation of As and Cd in rice grains through adsorption, transport regulation, and co-precipitation (Shao et al., 2017; Fleck et al., 2013). The selection of silicon carriers requires careful consideration of their dissolution kinetics and environmental impacts. Silicon-based soil additives, including calcium silicate, sodium silicate, and silica, exhibit differential efficacy in mitigating Cd accumulation in rice grains through distinct geochemical mechanisms. As a slow-release silicate fertilizer, calcium silicate undergoes progressive dissolution in the pedosphere, generating sustained fluxes of plant-available silicic acid (Si(OH)_4_) while concurrently releasing calcium ions (Ca^2+^). The liberated Ca^2+^ facilitates Cd immobilization through precipitation reactions (e.g., CdCO_3_ or Cd(OH)_2_ formation) and cation exchange processes, thereby reducing Cd^2+^ bioavailability in the rhizosphere. In contrast, sodium silicate demonstrates rapid dissolution characteristics, providing immediate silicon bioavailability. The mineralogical form of silica exhibits inherently lower solubility, functioning as a persistent silicon reservoir through slow weathering processes. One study showed that highly soluble sodium silicate resulted in a 26.4% reduction of Cd in seeds, while slow-release calcium silicate reduced Cd in seeds by 55.1%. Silica-containing materials such as silica, calcium silicate, and composites such as biochar and hemicellulose loaded with silica-containing compounds were also effective in reducing Cd accumulation in rice grains when applied to the soil. Therefore, adding different types of silica-containing materials into magnetically modified biochar can enhance the remediation capacity of the biochar for Cd (Sun et al., 2021; Leksungnoen et al., 2019). Thus, incorporating silicon-containing materials, such as calcium silicate, into magnetic biochar could enhance its ability to reduce Cd and As accumulation and alleviate growth inhibition caused by excessive iron. Chalmardi’s study shows that adding silicon fertilizer alleviates plant iron toxicity and promotes plant growth; silicon nutrition could ameliorate the harmful effects of Fe toxicity, possibly through the reduction of plant Fe concentration and improvement of antioxidant enzyme activity (Chalmardi et al., 2014). In view of this, silicon–iron-modified biochars were prepared to remediate rice soil contaminated by Cd and As simultaneously.

The accumulation of Cd and As in rice grains depends on their forms, which greatly affect the mobility and toxicity of Cd and As in the rhizosphere and are influenced by the soil’s physicochemical properties and microbial activities. Cd exists in water-soluble forms, such as CdCl_2_, Cd(NO_3_)_2_, and CdCO_3_, which can be easily absorbed by plants, while less available to rice in less mobile, insoluble forms such as Cd precipitates and colloidal Cd (Ma et al., 2020; Khaokaew et al., 2011). Similarly, As in the form of As(III) is more active and toxic than As(V) (Zhou et al., 2022).

Soil microbial communities play a crucial role in regulating metal speciation. Numerous studies have found that biochar can influence the Cd-As morphology transformation by affecting soil physicochemical properties and microbial activity. Weiwei’s study demonstrated that the addition of biochar increased soil porosity and contributed to the growth of As-oxidizing and As-methylating microorganisms, leading to significant increases in *aioA* and *arsM* gene abundance (Zhai et al., 2017). Herath’s study indicated that the addition of silica-rich biochar modulated the activity of *Geobacter* and *Bacillus* in rice soils, resulting in a significant increase in *aioA* gene abundance, approximately twice that of the blank control, which in turn affected the conversion of As from the more toxic As(III) to the less toxic As(V) (Herath et al., 2020). The addition of biochar to the soil influences soil pH and redox conditions, contributing to an elevated abundance of sulfate-reducing bacteria and a significant increase in *DsrB* gene expression(Chen et al., 2023). Additionally, iron oxides serve as electron acceptors, enhancing *SRB* activity and promoting *DsrB* expression. Silicon creates favorable conditions for microbial activity, while changes in soil pH and dissolved organic carbon further support microbiological activity (Tao et al., 2024). Similarly, As can be reduced from its less toxic form, As(V), to the more toxic form, As(III), through microbial action mediated by *arsC* genes. Additionally, As methylation and oxidation processes, mediated by genes such as *aioA* and *arsM*, serve as detoxification pathways that regulate As speciation and mobility in the soil (Rosen, 1999; Zou et al., 2022).

The incorporation of materials such as biochar, magnetite, and silica into soils has been demonstrated to significantly influence the speciation of heavy metals. Biochar-based additives primarily modify the soil’s chemical environment by altering parameters such as pH, redox potential, and dissolved organic carbon (DOC) concentrations, thereby affecting heavy metal speciation. Additionally, biochar serves as an excellent carrier for various soil microorganisms, promoting microbial activity and diversity. Under reductive conditions, the addition of biochar to paddy soils has been shown to enhance the abundance of microbial genes, such as *arrA* and *arsC*, which may contribute to the increased release of As in paddy soils (Yang et al., 2018).

Studies have shown that the mixed application of raw straw biochar and oyster shells led to a significant reduction in the flow fraction of As (III) and As (V) in soils heavily contaminated with arsenic (Chen et al., 2018). Zheng et al. (2022) found that chitosan-EDTA-modified biochar can effectively reduce the solubility and bioavailability of Cd, thereby mitigating its potential harm to plants and the environment. Similarly, magnetite and silicate materials can migrate the speciation of heavy metals by forming precipitates or complexes. Magnetite can effectively remediate anionic arsenic (As) heavy metals through electrostatic adsorption and surface complexation. Additionally, Fe(III) can easily complex with the hydroxyl groups in paddy soil to form iron hydrate complexes on the biochar surface, which can absorb As(III) (Herath et al., 2020). Iron oxides can also transform water-soluble Cd^2+^ into a more stable form by adsorption or complexions, thereby reducing the bioavailability of Cd (Ma and Qiao, 2024). As for silicon-based materials, studies have found that water-soluble silicon in soil solutions can form structurally complex polysilicic acid gels through polymerization, which can combine with heavy metal ions (M) to form M–Si complexes that plants cannot absorb, thereby reducing the bioavailability of Cd (Belton et al., 2012).

Herath et al. (2020) found that silicon-modified biochar can effectively immobilize more mobile As(III) in the rhizosphere by reducing Fe(III) to Fe(II), adsorbing Fe(III) through silicates, chelating Fe(III) with hydroxide ions, and forming a silicon (Si) intermediate layer on the biochar’s surface.

Despite the growing interest in using modified biochar for soil remediation, research on the comprehensive effects of composite materials on heavy metal speciation remains limited. The majority of studies have focused on the role of biochar in reducing heavy metal bioavailability and altering the physicochemical properties (Yang et al., 2021; Mohan et al., 2007). However, there has been a lack of research into how these materials affect the speciation of heavy metals and the underlying physicochemical and microbial mechanisms that control the transformation of heavy metals in soils.

The objectives of this study are as follows: (1) to prepare biochars by incorporating three different silicates—calcium silicate, sodium silicate, and silica dioxide—along with magnetite; and (2) to evaluate the effects of these pristine and silicon–iron magnetic biochar composites on the migration and speciation of cadmium (Cd) and arsenic (As), as well as their influence on biotransformation-related functional genes in soil. By elucidating the biogeochemical mechanisms underlying the impact of silicon–iron biochar modifications on Cd and As dynamics, this study aims to provide novel insights into the immobilization and stabilization of these elements within soil systems.

## 2 Materials and methods

### 2.1 Material production

The pristine biochar was prepared from agricultural waste—rice straw. The magnetic biochar was then prepared through a co-precipitation method involving the raw biomass material (rice straw) and magnetite, according to Wu et al. (2018).

Sodium silicate (Na_2_SiO_3_), calcium silicate (CaSiO_3_), and silicon dioxide (SiO_2_) with varying mass ratios of 5 and 10% were added to the materials. The freeze-dried biomass was subsequently subjected to pyrolysis in a muffle furnace (SGM-M6/10, China) at 700°C under a nitrogen (N_2_) atmosphere for 2 h. The resulting biochar was ground and sieved to achieve a particle size of less than 1 mm, and it was labeled as 5% SMBC1, 10% SMBC1, 5% SMBC2, 10% SMBC2, 5% SMBC3, and 10% SMBC3. The percentages of 5 and 10% indicate the mass concentration of the silicon-based material, while SMBC1-3 indicates the addition of sodium silicate, calcium silicate, and silicon dioxide into the materials, respectively. BC indicates the pristine biochar.

### 2.2 Soil preparation

Paddy soil was collected from fields contaminated with Cd-As composites in the Shangyu District of Shaoxing City, Zhejiang Province, China. The top 0–20 cm layer of soil was collected and air-dried. It was then ground through a 2-mm sieve and stored for later use. The basic physicochemical properties of the tested soil were analyzed (Table 1), including pH, DOC, the available concentrations of silicon, and the available concentrations of Cd and As in the soil.

**Table 1:** Physicochemical properties of the tested soil.

pH	DOC (mg·kg^−1^)	Total Cd concentration (mg·kg^−1^)	Total As concentration (mg·kg^−1^)	Sand particle (%)	Silt particle (%)	Clay particle (%)
6.24	214.52	0.98	71.72	30.77	58.37	10.85

### 2.3 Soil incubation experiment

From August to December 2023, an 84-day soil culture experiment was conducted in a biochemical laboratory incubator in Hangzhou, Zhejiang Province, China. Then, 1 kg of soil was thoroughly mixed with additives (biochar, BC, and modified biochar, SMBC) at a 1% mass ratio. This mixture was added to plastic cans, deionized water was included, and the materials were mixed well with the soil for dry–wet alternation (DW).

The DW management group underwent three alternating dry–wet treatments during the testing period. In each cycle, the field water-holding capacity was maintained at 100%, followed by the addition of pure water to create a flooding depth of 2–3 cm for 14 days. The lids were then opened to facilitate natural drying until the moisture level reached 60% of the field water capacity. Throughout this 14-day drying cycle, water was measured and replenished every 3 days to ensure that the field water-holding capacity remained at 60%. Beginning from the fifth DW cycle (Day 56), the soil water concentration was preserved at 60% of the field water capacity to simulate the dry period during the rice grain filling stage. The perforated lids were subsequently placed in a biochemical incubator at a constant temperature of 25°C. The treatments were labeled as follows: control (no additive addition), BC (pristine biochar), 5% SMBC1, 10% SMBC1, 5% SMBC2, 10% SMBC2, 5% SMBC3, and 10% SMBC3, with three replicates for each. The percentages indicate the mass concentration of the silicon-based material, while SMBC1-3 corresponds to the addition of silicate, calcium silicate, and silicon dioxide, respectively.

### 2.4 Sampling and measurements

#### 2.4.1 Physicochemical properties

On the 0th, 7th, 21st, 35th, 49th, 63rd, 77th, and 84th days of soil culture, 50 g of soil was collected and dried through 100 and 10 mesh sieves.

Soil pH (soil–water ratio 1: 2.5) was measured using a pH meter (Seven Excellence Cond Meter S700, Mettler Toledo, Switzerland). Soil DOC concentration (soil–water ratio 1:5, leaching for 1 h) was assessed using a TOC analyzer (TOC-L CPN, Shimadzu, Japan).

Cd and As were extracted using 0.01 mol·L^−1^ CaCl_2_ (1:30 soil-to-water ratio, 2 h) and 0.05 mol·L^−1^ NH_4_H_2_PO_4_ (1:25 soil-to-water ratio, 16 h) for the measurement of proximate bioavailability, respectively. The concentrations of Cd and As were determined using a graphite furnace atomic absorption spectrophotometer (AA-7000, Shimadzu, Japan) and a two-channel atomic fluorescence photometer (AFS-2202E, Beijing Han Guang, China), respectively. X-ray photoelectron spectroscopy (XPS, Thermo Scientific K-Alpha, USA) was used to analyze the valence states and forms of Cd and As in the soil on Day 7, as well as the valence states and forms of Fe, Si, Cd, and As in the recovered SMB in soil on Day 7.

#### 2.4.2 Speciation of Cd and As

Soil samples collected on the 7th, 21st, 63rd, and 77th days were used to measure the speciation of Cd and As. Continuous extraction of soil Cd was conducted using the improved BCR method, with the specific extraction process and operations shown in Table 2. The Cd concentration was determined using a graphite furnace atomic absorption spectrophotometer (AA-7000, Shimadzu, Japan).

**Table 2:** BCR sequential extraction of Cd.

Soil Cd speciation	Procedures
Weak acid soluble state	20 mL 0.11 mol CH_3_COOH, 16 h
Reduced state	20 mL 0.5 mol NH_2_OḤHCl (2 M HNO_3_ acidification, pH 2), 16 h
Oxidizable state	5 mL 8.8 mol H_2_O_2_ (pH 2–3, HNO_3_ acidification), 1 h
	5 mL 8.8 mol H_2_O_2_ (pH 2–3, HNO_3_ acidification), 1 h
	20 mL 1 mol NH_4_OAc (pH 2, HNO_3_ acidification), 16 h
Residue state	1 HNO_3_: 3 HCl (Leave overnight)
	2 mL HClO_4_ (180°C, 2 h) digestion

A total of 0.2 g of freeze-dried soil samples (100 mesh) was weighed in a polypropylene centrifuge tube. Phosphoric acid (10 mL, 2%; volume ratio 2:98) was added as an extraction agent and mixed evenly. The species of As, including As(III), As(V), MMA(V), and DMA(V), were analyzed using HPLC-ICP-MS (Shimadzu LC-20AB, Agilent 7900 ICP-MS, USA).

#### 2.4.3 Measurements of functional genes

Soil samples were collected on the 7th, 21st, 63rd, and 77th days, with 0.20 g of each sample accurately weighed. The soil samples were then freeze-dried and stored at −80°C. Total microbial DNA was extracted using a DNeasy Power-Soil DNA Kit according to the manufacturer’s protocol. The abundance of sulfate-reducing genes (*DsrB*) and three types of As functional genes, arsenite oxidase (*aioA*), arsenate reductase (*arsC*), and arsenite methyltransferase (*arsM*), were quantified by real-time PCR (Quéméneur et al., 2010). The primers and qPCR sequences used are presented in Table 3. Conventional PCR amplification of *arsC*, *arsM*, *aioA*, and *DsrB* genes was conducted using the primers *arsC-F*/*arsC-R*, *arsM-F*/*arsM-R*, *AroAdeg2F*/*AroAdeg2R*, and *DSR-p2060F*/*DSR-4R* (Walker, 2001; Zhang et al., 2015; Lin et al., 2018). Detection was provided by Shanghai Personal Biotechnology Co., Ltd. PCR sequencing was conducted using the BigDye Terminator V3.1 Cycle Sequencing Kit (BDT 3.1, ABI, USA) on a PCR instrument (EDC-810, Beijing Dongsheng Innovation Biotechnology Co., Ltd). The PCR products were further subjected to purification. The pooled DNA products were then used to construct an Illumina paired-end library before sequencing (2 × 250) on an Illumina MiSeq platform (Shanghai Personal Biotechnology Co., Ltd). The gene expression data used in this study were obtained from Figshare,^1^ which contains qPCR data for soil Cd and As-related genes under different treatment conditions.

**Table 3:** Primers and qPCR sequence information.

Target gene	Primer	Sequences	References
*aioA*	*AroAdeg2F*	GTCGGYTGYGGMTAYCAYGYYTA	Pipattanajaroenkul et al. (2021)
	*AroAdeg2R*	YTCDGARTTGTAGGCYGGBCG	
*arsM*	*arsM-F*	TCYCTCGGCTGCGGCAAYCCVAC	Zhang et al. (2015)
	*arsM-R*	CGWCCGCCWGGCTTWAGYACCCG	
*arsC*	*arsC*-F	TCGCGTAATACGCTGGAGAT	
	*arsC*-R	ACTTTCTCGCCGTCTTCCTT	
*DsrB*	*DSR-p2060F*	CAACATCGTYCAYACCCAGGG	Du and Behrens (2024)
	*DSR-4R*	GTGTAGCAGTTACCGCA	

### 2.5 Statistical analysis

The means of different treatments were compared using a one-way ANOVA followed by Tukey’s test at a significance level of *p* < 0.05. Additionally, Pearson’s correlation analysis was performed. All statistical analyses were conducted using IBM® SPSS Statistics 27.0 and Origin 2021 software.

## 3 Results

### 3.1 Dynamics of soil pH and DOC

**Figure 1:**
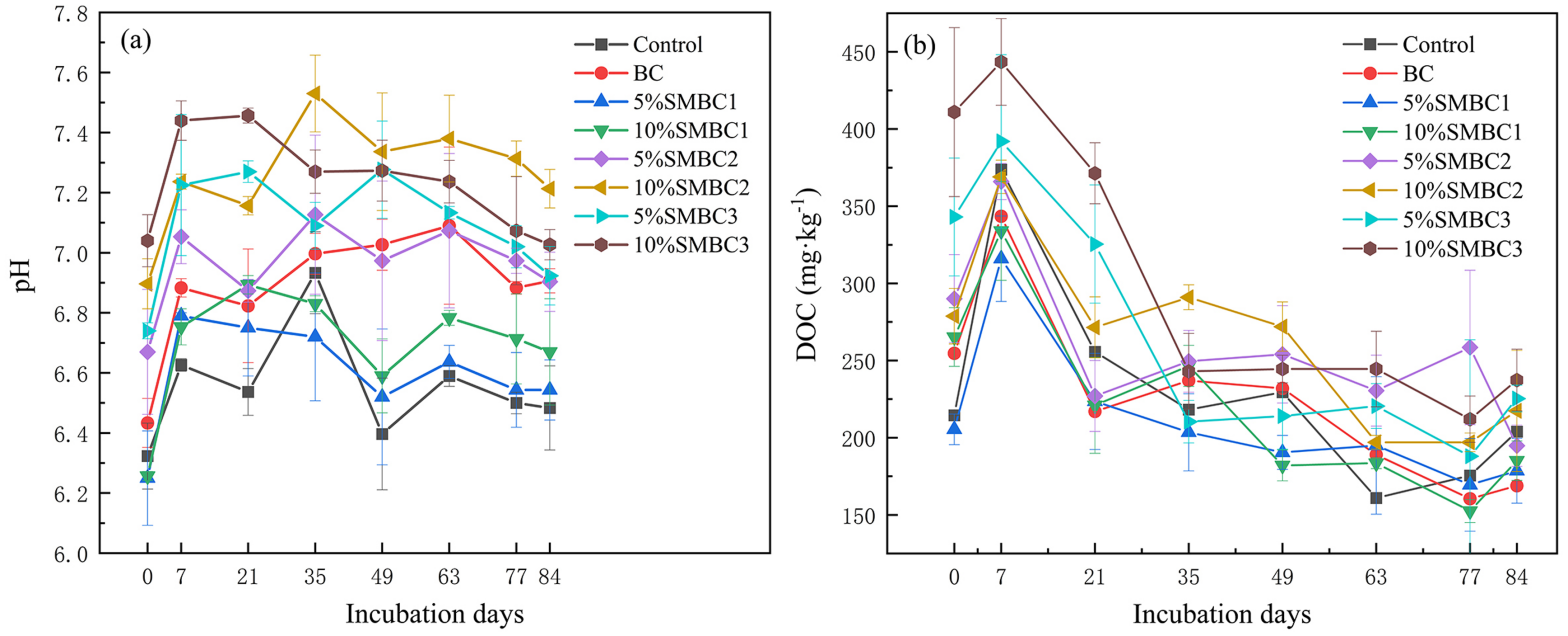
Changes in pH **(a)** and DOC **(b)** concentration during the soil culture period. The treatments were labeled as follows: Control (no additives addition), BC (pristine biochar), and SMBC (silicon–iron-modified biochar). The percentages indicate the mass concentration of the silicon-based material, while SMBC1-3 refers to the addition of silicate, calcium silicate, and silicon dioxide, respectively. The same applies below.

During the incubation cycle, the soil pH initially increased before stabilizing, peaking on day 35, followed by a decline and subsequent leveling off (Figure 1). Compared to the control, the addition of BC and SMBC significantly increased soil pH (*p* < 0.05), with increases ranging from 6.43 to 6.90 and from 6.89 to 7.21, respectively. Among the treatments, calcium silicate caused the greatest increase in soil pH, followed by silicon dioxide, while sodium silicate had the least impact. Higher application rates of silicon-containing materials further elevated soil pH levels, with the 10% SMBC2 treatment showing the most significant increase. Water management also influenced pH, with flooded conditions exhibiting significantly higher pH levels compared to dry conditions. Additionally, As(III) showed a strong negative correlation with pH, suggesting that biochar additives effectively increased soil pH while reducing As(III) concentrations.

DOC plays a crucial role in regulating the mobility of As and Cd in the soil. The results showed that the addition of additives caused the DOC concentration to initially increase before decreasing. Compared to the control, both BC and SMBC significantly enhanced soil DOC concentration (Figure 1a) by 6.6–16.39%. During the incubation period, DOC concentrations increased significantly from day 0 to day 7, reaching a peak on day 7 before declining. By the end of the incubation, compared to the control, the addition of BC, 5% SMBC1, 10% SMBC1, and 5% SMBC2 significantly reduced DOC concentrations by 17.18, 12.42, 9.23, and 4.47%, respectively (*p* < 0.05). In contrast, the SMBC3 treatment significantly increased DOC concentrations. Among all treatments, BC resulted in the lowest soil DOC concentration, with a reduction of 17.18%.

### 3.2 Changes in Cd and As speciation in paddy soil

#### 3.2.1 Speciation changes of Cd in paddy soil

**Figure 2:**
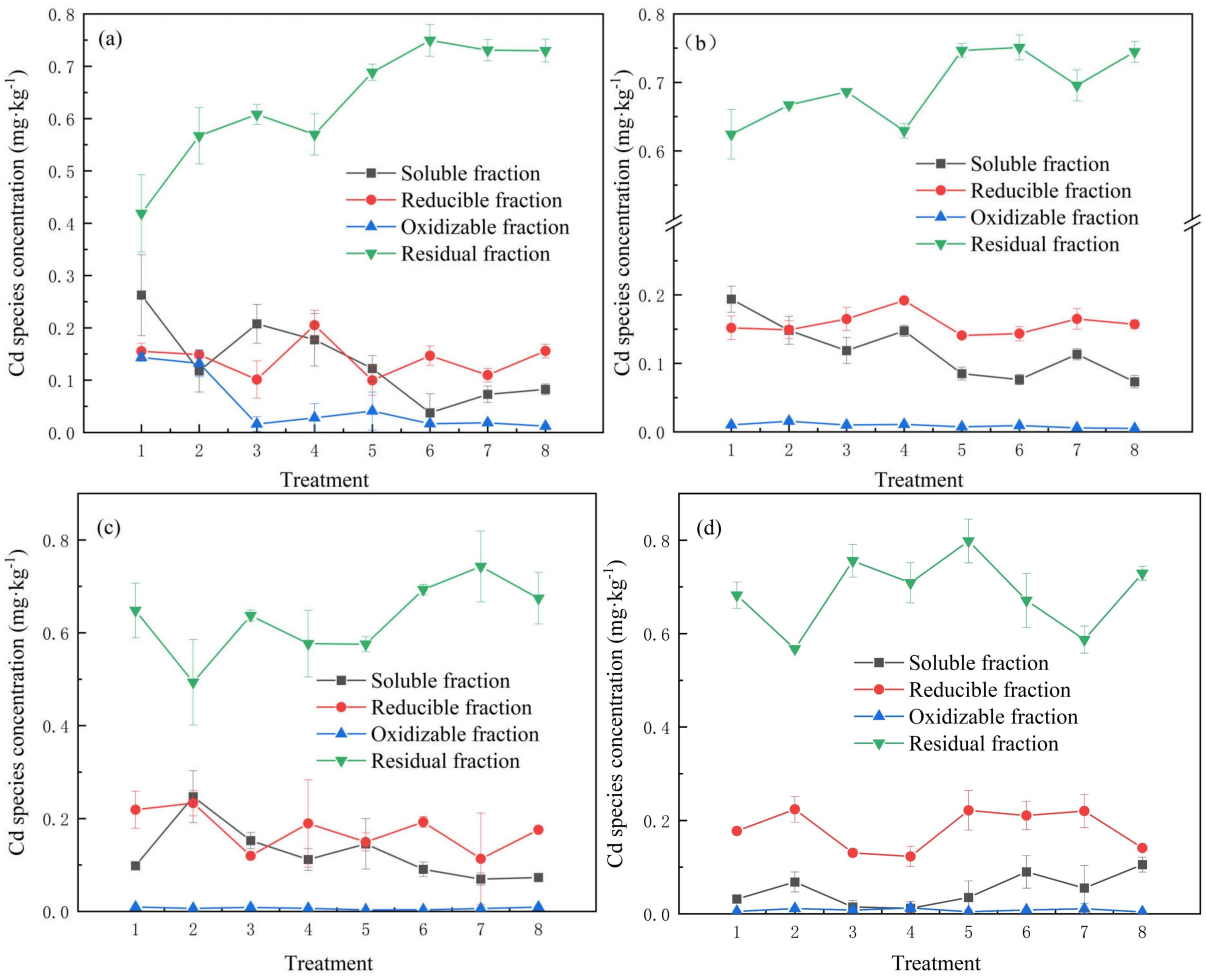
Concentration of various forms of Cd in soil on days 7 **(a)**, 21 **(b)**, 63 **(c)**, and 77 **(d)**. 1. Control; 2. BC; 3. 5% SMBC1; 4. 10% SMBC2; 5. 5% SMBC2; 6. 10% SMBC2; 7. 5% SMBC3; 8. 10% SMBC3. Error bar = ± standard deviation (*n* = 3). The same applies below.

As shown in Figure 2, at four different time points during the incubation, Cd was primarily in the residual state, with concentrations ranging from 0.42 mg·kg^−1^ to 0.73 mg·kg^−1^. The addition of SMBC promoted Cd stabilization by increasing its residual form and reducing its oxidizable and soluble forms throughout the incubation period. Compared to the control, SMBC treatments significantly (*p* < 0.05) decreased soluble Cd concentrations by 26.31–63.15% and increased residual Cd concentrations by 2.94–16.17%, with 5% SMBC2 achieving the highest increase of 16.17%. In contrast, BC increased soluble Cd concentrations and reduced residual Cd concentrations during aerobic incubation. The effects of BC and SMBC on Cd transformation varied under different moisture conditions. Under flooded conditions, SMBC treatments significantly increased residual Cd concentrations by 36.58–80.48%, while under dry conditions, and the increase ranged from 8.01 to 19.23%.

#### 3.2.2 Speciation changes of As in paddy soil

**Figure 3:**
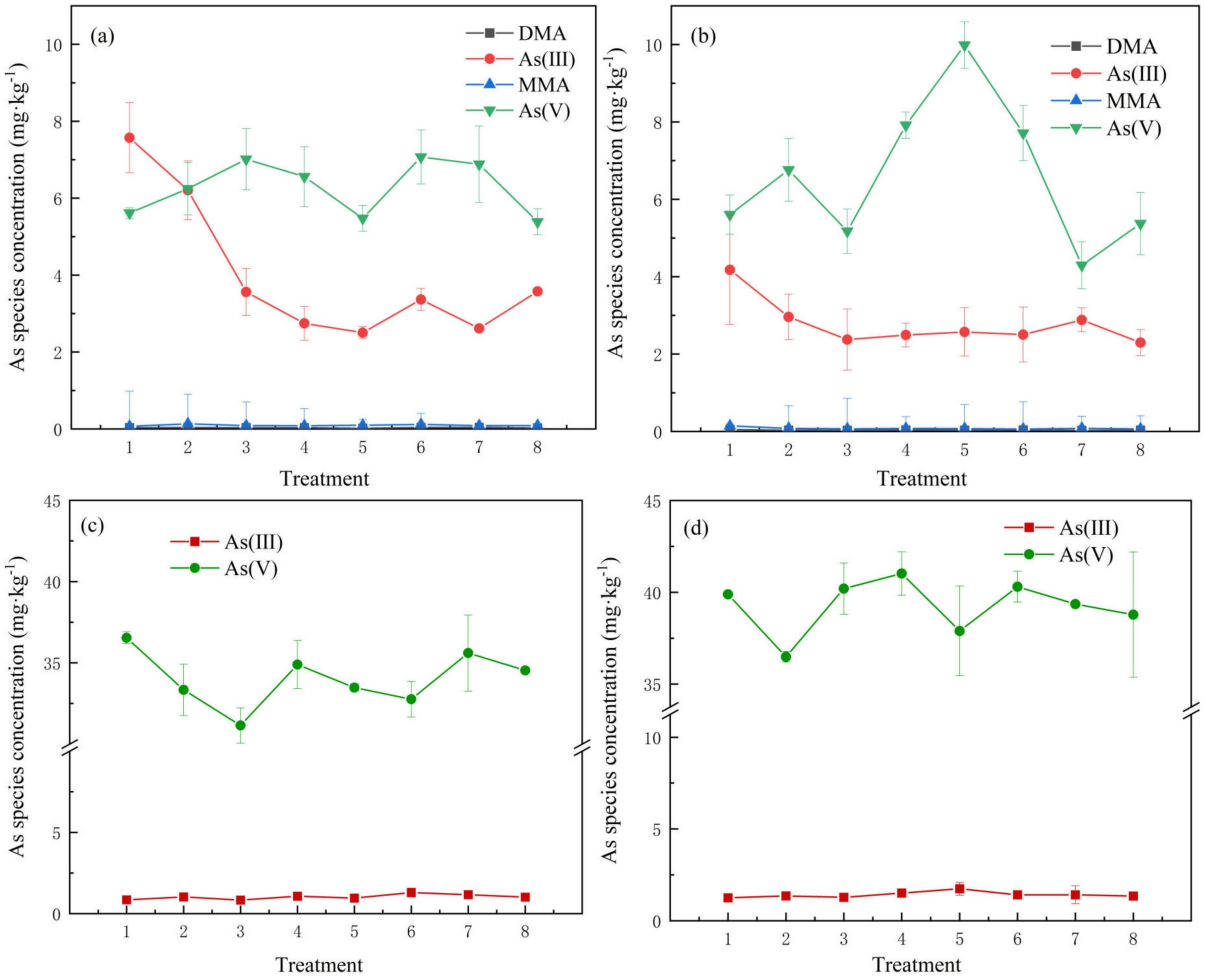
Speciation changes of As in the soil on days 7 **(a)**, 21 **(b)**, 63 **(c)**, and 77 **(d)**. Error bar = ± standard deviation (*n* = 3).

**Figure 4:**
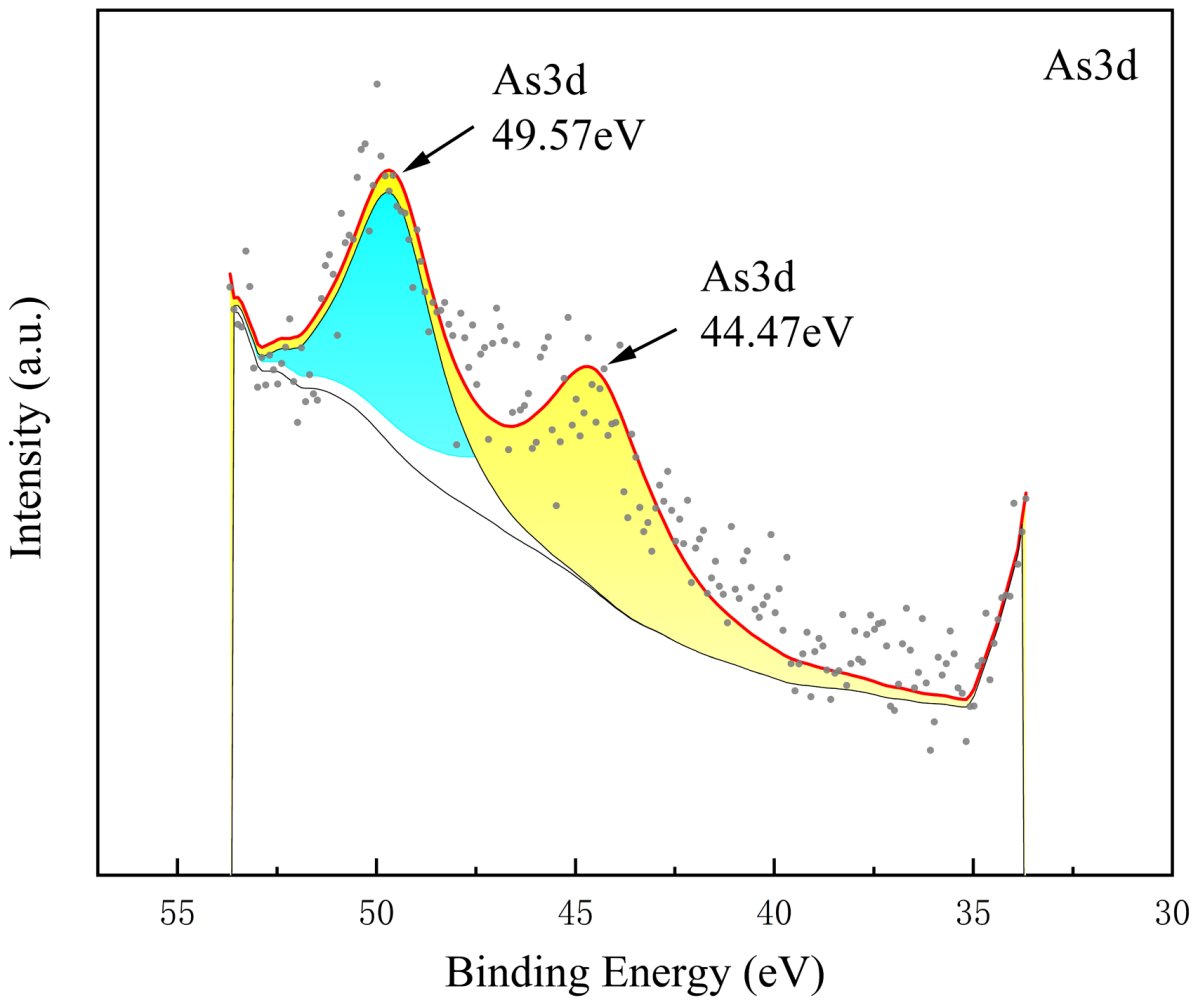
Illustration of the As*3d* spectra of the soil on Day 7. The *3d* peak corresponding to adsorbed As(III) is observed at 44.47 eV.

Compared to the control group, the addition of SMBC significantly increased the concentration of As(V) by 11.42–26.07% (*p* < 0.05) and decreased the concentration of As(III) by 18.09–66.97% (*p* < 0.05) (Figure 3). The effect of SMBC on the transformation of As species in the soil varied under different water management conditions. During the preculture period under flooded conditions, the concentration of As(III) was higher than that of As(V), leading to the reduction of As(V) to As(III). Conversely, under dry conditions, As(III) was transformed into As(V), resulting in a significant increase in As(V) concentration; however, changes in organic As levels were negligible. On day 7 of incubation under flooded conditions, the concentration of As(V) in the 5% SMBC2 treatment was 5.47 mg·kg^−1^. By day 21, under aerobic conditions, this concentration had significantly increased by 45.19%. In the later stages of soil cultivation, as the soil dried on days 63 and 77, As(III) was largely converted to As(V), with no significant differences observed among treatments in terms of As(V) levels. The concentrations of As(V) in treatments with 5% SMBC added were 39.65 mg·kg^−1^ and 30.86 mg·kg^−1^. On the 7th day of incubation, As(III) and As(V) was detected in the As 3d spectrum by X-ray photoelectron spectroscopy (XPS) analysis of the soil (Figure 4), with characteristic peak positions at 44.47 eV and 49.57 eV, respectively, consistent with standard reference values. These results are consistent with the As speciation observed under waterlogged conditions. The primary binding forms of As(V) in the soil were identified as iron arsenate complexes and silica-adsorbed As (Lee et al., 2024).

### 3.3 Variation of bioavailable Cd and As

**Figure 5:**
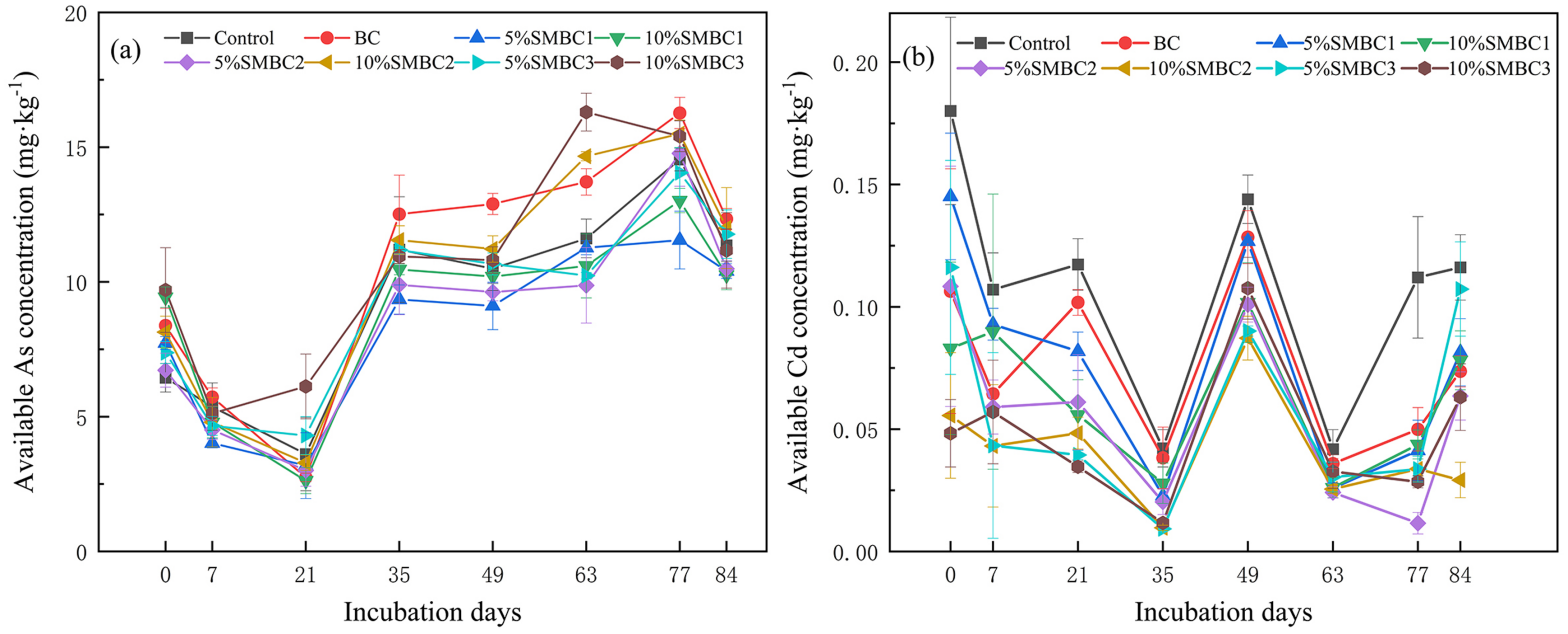
Dynamic changes in bioavailable As **(a)** and Cd **(b)** concentrations. Error bars = ± standard deviation (*n* = 3).

The bioavailable concentration of As in each soil treatment showed a trend of initially decreasing and then increasing(Figure 5). The soil bioavailable As concentration decreased from 0 to 21 days, significantly increased from 21 to 35 days, and subsequently decreased after a slow rise between days 35 and 77. At the end of the incubation period, the addition of 5% SMBC1 and SMBC2 reduced the bioavailability of As by 8.37 and 7.66%, respectively (*p* < 0.05). Similarly, the addition of 10% SMBC1 and SMBC3 lowered the As concentration by 9.69 and 1.67%, respectively (*p* < 0.05). The bioavailable As concentrations with the addition of 5% SMBC3 and 10% SMBC2 did not increase significantly. Compared to the control, the 10% SMBC1 treatment resulted in the lowest soil bioavailable As concentration of 10.25 mg·kg^−1^.

During the incubation period, bioavailable Cd concentrations initially decreased, peaking on day 49 (in a dry state) before gradually declining. In the final wet-dry cycle, bioavailable Cd levels increased but remained below the initial concentration. The application of SMBC significantly reduced soil Cd concentrations. By the end of the incubation period, treatments with 5% SMBC1, SMBC2, and SMBC3 reduced Cd concentrations by 26.36, 42.72, and 9.09%, respectively (*p* < 0.05) compared to the control. Similarly, 10% SMBC1, SMBC2, and SMBC3 reduced Cd concentrations by 29.09, 73.63, and 42.72%, respectively (*p* < 0.05). Among all treatments, 10% SMBC2 achieved the most significant reduction, with a bioavailable Cd concentration of 0.029 mg·kg^−1^.

### 3.4 Changes in the abundance of As and Cd-related functional genes

**Figure 6:**
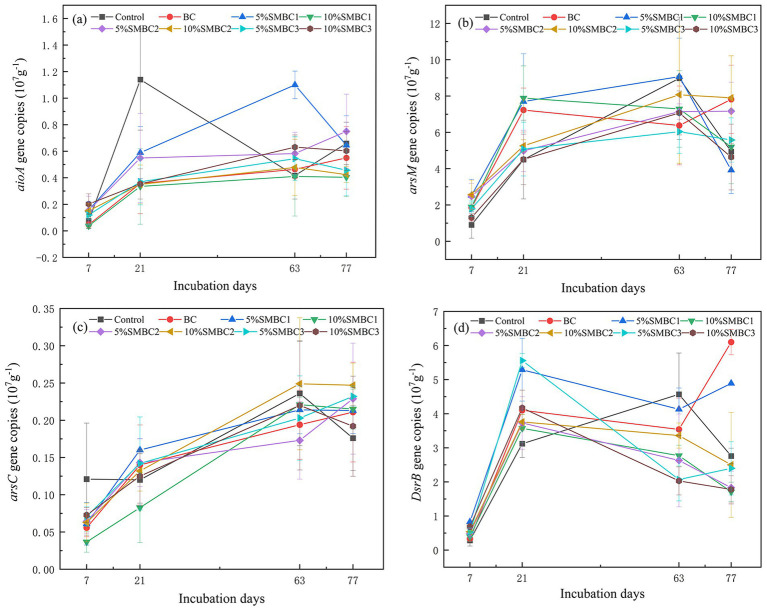
Changes in the abundance of *arsC* **(a)**, *aioA* **(b)**, *arsM* **(c)**, and *DsrB* **(d)** genes during soil culture. Error bar = ± standard deviation (*n* = 3).

The abundance of functional genes in each treatment exhibited an increasing trend throughout the culture period (Figure 6). Compared to the control, the addition of SMBC significantly enhanced the abundance of the arsenite oxidase (*aioA*) and arsenite methyltransferase (*arsM*) genes (*p* < 0.05). Notably, the addition of SMBC2 resulted in a higher abundance of the *arsM* gene in comparison to other treatments, with 5% SMBC2 and 10% SMBC2 increasing the *arsM* gene abundance by 45.14 and 59.91%, respectively. On day 63 of the culture, the abundance of the *aioA* gene in the 5% SMBC1 treatment was approximately twice that of the control (Figure 6a). At the end of the cultivation period, the concentration of the *aioA* gene in the treatment with 5% SMBC2 increased by 15.38% compared to the control, while gene copies in all other treatments decreased. Additionally, changes in the abundance of functional genes across treatments were linked to water management conditions, with the abundance of the *DsrB* gene being significantly higher under aerobic conditions than in waterlogged conditions.

On Day 77, the abundance of the *arsM* and *arsC* genes was highest in the 10% SMBC2 treatment, with values of 7.90 × 10^7^ copies per gram of dry material and 0.24 × 10^7^ copies per gram of dry material, respectively (Figures 6b,c). Compared to the control, the abundance of the *arsC* gene did not change significantly, while the 10% SMBC2 treatment exhibited the highest abundance. Conversely, the abundance of the *DsrB* gene decreased following the addition of materials, with the 10% SMBC1 treatment showing the lowest abundance at 1.71 × 10^7^ copies per gram of dry material.

## 4 Discussion

### 4.1 Effect of additives on soil physical and chemical properties

The addition of SMBC increases soil pH due to the hydrolysis of alkaline substances in biochar, which increases the concentration of hydroxide ions (OH^−^) in the soil. Moreover, SMBC not only contains alkaline substances but also produces OH^−^ through the hydrolysis of silicate ions (SiO_3_^2−^) to further enhance soil pH (Li et al., 2017).

At the end of the incubation period, the addition of SMBC3 increased the DOC concentration in the soil compared to the control, as biochar, being carbon-rich, releases various organic molecules into the soil (Liu et al., 2019). In addition, biochar creates a favorable growth environment for soil microorganisms. During the decomposition and transformation of organic substances, these microorganisms produce metabolites, including soluble organic carbon. SMBC3 significantly contributed to the increase in DOC concentration because sodium silicate significantly increased soil pH levels and promoted the mineralization of dissolved organic matter. Conversely, the addition of SMBC1 and SMBC2 reduced soil DOC concentrations. This reduction was attributed to the formation of complexes between DOC molecules and silica surfaces, which decreased their solubility in the soil solution. The silica-loaded surface of SMBC exhibits relatively stable chemical properties, minimizing its impact on soil DOC concentrations (Rahimzadeh and Ghassemi-Golezani, 2024). Additionally, Ca^2+^ from calcium silicate (CaSiO_3_) can adsorb onto negatively charged DOC molecules through electrostatic interactions, as carboxylic acids, phenols, and other functional groups in DOC typically carry negative charges. This adsorption reduces DOC solubility, leading to the precipitation or fixation of Ca^2+^ onto soil particle surfaces (Song et al., 2023). Furthermore, no significant differences in Eh values (*p* > 0.05, data not shown) were observed among treatments during the incubation period.

### 4.2 SMBC migrates Cd and As speciation in soil

#### 4.2.1 Cd speciation changes

SMBC migrated Cd speciation by altering soil physicochemical properties, forming precipitates, and creating complexes. The increase in soil pH facilitates the formation of insoluble silicate precipitates through the reaction of silicate ions with Cd. Moreover, studies have demonstrated that silicon can alter the chemical form of Cd in alkaline soil solutions. In the present study, the concentrations of reducible and residual Cd have increased.

**Figure 7:**
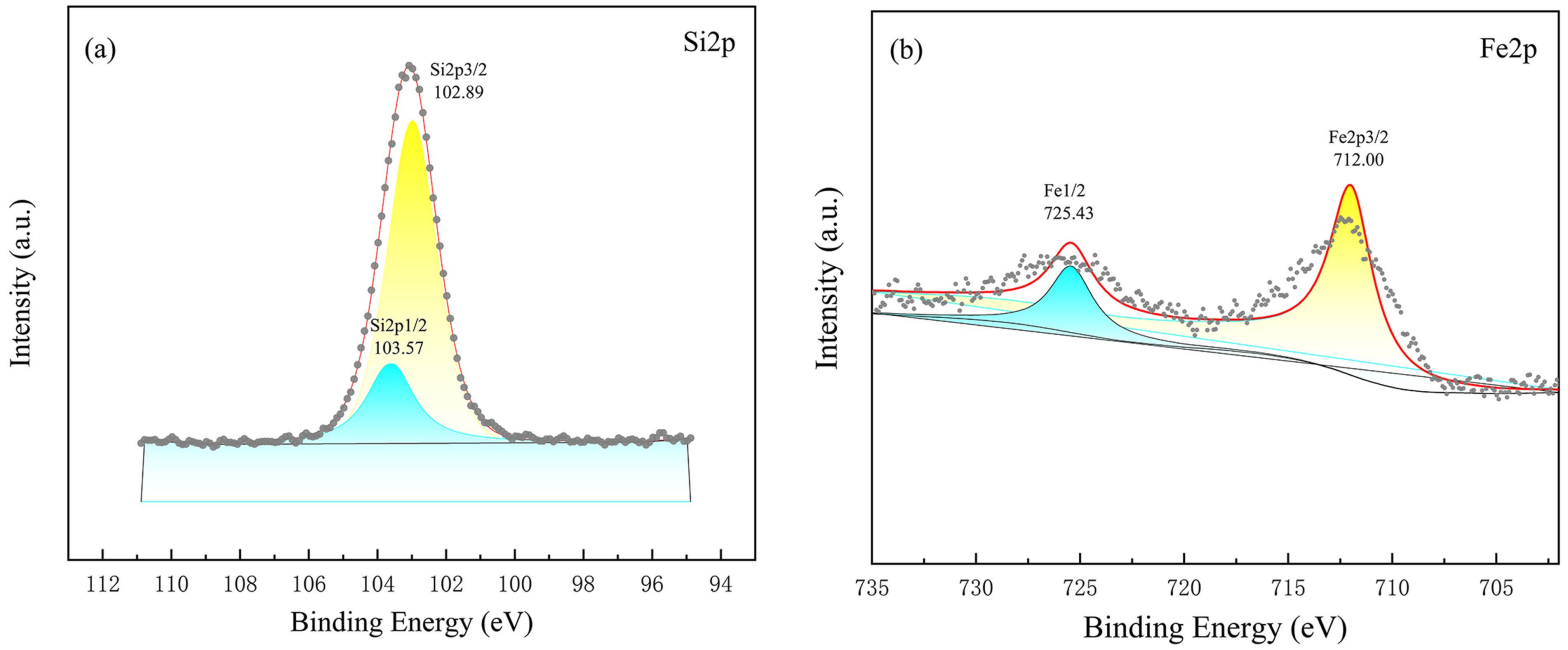
XPS spectra of Si *2p* **(a)** and Fe *2p* **(b)** for silicon-iron modified biochar recovered from the soil.

After soil incubation, the XPS spectra of the recovered materials (Figure 7) reveal photoelectron peaks for Fe 2*p* and Si 2*p*. The Fe present in the hybrid orbitals Fe *3d* 3/2 and Fe *3d* 1/2 predominantly exists as Fe_3_O_4_ and FeOOH. The Si 2*p* peaks are observed at binding energies of 102.89 eV and 103.57 eV, indicating that Si exists in a single chemical state, primarily in a tetrahedral configuration. This not only enhances the stability of iron oxides—maintaining their strong adsorption capacity—but also forms Fe–O–Si bonds, which further stabilize the combined structure. The interaction between silicon and Cd leads to Cd binding to the Si–O group, resulting in the formation of the Si–Cd complex (Li et al., 2017). This complex is not water-soluble, thus reducing the bioavailability of Cd (Furuya et al., 2016). Additionally, iron oxides in the soil may reduce soluble iron ions (Fe^2+^), which can react with sulfides to form iron sulfide precipitates, further promoting the precipitation of Cd (Grüter et al., 2017).

The changes in functional genes also play a significant role in the migration of Cd speciation. Sulfur is an essential nutrient in paddy soil, and the redox cycle that occurs during the flooding and drainage of rice fields has an important influence on the activity of metals (Zhou et al., 2024). *DsrB* is a key functional gene responsible for sulfate reduction in sulfate-reducing bacteria. It encodes an enzyme that plays a vital role in sulfate reduction and catalyzes the reduction of SO_4_^2−^ to sulfide S^2−^, which can form metal sulfide precipitates with various heavy metal ions, thereby immobilizing these metal ions (Anantharaman et al., 2018). No obvious S and Cd peaks were detected in the XPS spectrum (Supplementary Figure S1), but the concentration of SO_4_^2−^ decreased from 142.4 mg·kg^−1^ to 116.8 mg·kg^−1^ in the 5% SMBC1 treatment on Day 7 compared to CK, which further confirmed the formation of S^2−^ and CdS after the addition of SMBC.

The addition of SMBC upregulated the expression of the *DsrB* gene, which may be attributed to several factors. First, iron oxides can serve as electron acceptors in the sulfate reduction process, promoting the growth and activity of sulfate-reducing bacteria (*SRB*) (Kwon et al., 2014). The addition of iron oxides may provide more electron acceptors for *SRB*, thereby stimulating the expression of the *DsrB* gene. Second, silicon can increase the porosity of the soil, creating a more suitable environment for microorganisms, which in turn promotes the expression of the *DsrB* gene (Verma et al., 2022). Third, materials can indirectly influence microbial activity by altering soil pH and DOC concentration (Andersson and Nilsson, 2001). When the pH increases, the concentration of dissolved organic carbon (DOC) also tends to rise. The pH has an impact on the charge density of humic substances, and an increase in pH can stimulate microbial activity (Andersson and Nilsson, 2001). The addition of iron oxides and silicon may alter soil environmental conditions and affect the activity of *SRB* and the expression of the *DsrB* gene (Kirsten et al., 2021). A strong negative correlation was observed between *DsrB* expression and bioavailable As, suggesting that higher concentrations of bioavailable As may suppress the activity of *SRB*. The reduction in bioavailable As concentrations induced by SMBC likely alleviates this suppression, promoting the activity and abundance of *SRB* and subsequently leading to an increase in *DsrB* expression (Chen et al., 2020). After the incubation, the abundance of the *DsrB* gene in the SMBC1 treatment significantly increased, showing a favorable effect compared to calcium silicate and sodium silicate. This may be because silicon dioxide altered the chemical properties of the soil, indirectly affecting the activity of *SRB* (Kibangou et al., 2021).

#### 4.2.2 As speciation changes

Soil pH, redox potential, organic matter concentration, microbial activity, and the presence of iron and manganese oxides significantly influence the speciation transformation of As. During the soil cultivation process, the proportion of As(III) initially decreases due to the dry–wet (DW) cycle. Under aerobic conditions, As(III) undergoes oxidation, resulting in the conversion of As(III) to As(V), which has a strong specific affinity for iron, aluminum, and manganese oxides, allowing it to adsorb onto minerals in soil and sediments. As cultivation progresses, the addition of soil microbial biomass carbon (SMBC) treatments leads to an increase in As(V) concentrations, consistent with findings by Health et al. The transition from anaerobic to aerobic conditions results in a decrease in As(III) concentrations and an increase in As(V) concentrations. In the recycled materials, the X-ray photoelectron spectroscopy (XPS) spectra of As*3d* are shown in Supplementary Figure S2, indicating the complexation of As(V) with iron oxides (As*3d at* 49.99 eV).

Microbial activity plays a critical role in causing changes in As speciation. The microbial oxidation of As(III) is mediated by arsenite oxidase (*Aio*), which was strongly expressed under aerobic conditions (Days 21, 63, and 77; Eh = 614.24 ± 15.13 mV). A quantitative analysis of As-related functional genes confirmed that the arsenite oxidase gene (*aioA*) plays a crucial role in As(III) oxidation in biochar-amended soil (Sun et al., 2008). The addition of silicon–iron-modified biochar (SMBC) significantly increased the relative abundance of *aioA*-harboring microbiota. This incorporation of SMBC also significantly increased the relative abundance of *aioA*-containing microbiota. The iron oxides in the modified biochar served as additional electron acceptors and donor sites for microbial redox reactions, enhancing the activity and metabolic efficiency of these functional microorganisms. Additionally, this enrichment can be attributed to the porous structure and abundant surface functional groups of straw-derived biochar. The surface properties of the biochar provided excellent colonization sites for As-oxidizing microorganisms, thereby facilitating the oxidative transformation of As. In SMBC-amended soils, the relative abundance of the *aioA* gene increased significantly, showing a strong negative correlation with As(III) concentrations (*p* < 0.01), which is consistent with the findings of Yang et al. (2020). Since no significant differences (*p* > 0.05) were observed in Eh values across all treatments during incubation, the change in *aioA* gen abundance was caused by the addition of SMBC. This increase was facilitated by both the iron oxides and silicon components within biochar, in conjunction with As-oxidizing microorganisms indigenous to the soil. The micropores on the biochar surface could function as intermediate electron transfer mediators between microorganisms and iron oxides, thereby facilitating As(III) oxidation (Herath et al., 2020). The addition of biochar led to a significant enrichment of *aioA*-containing microorganisms, as the surface characteristics of the biochar provided favorable colonization sites for these As-oxidizing populations to facilitate oxidative transformation. In addition, Yang et al. found that *Nitrospira* is a major carrier of *aioA* and plays a crucial role as an As(III) oxidant (Yang et al., 2020). Other studies have also shown that nitrate reduction can be combined with As(III) oxidation, significantly enhancing the abundance of the *aioA* gene, especially by increasing the relative abundance of operational classification units (OTUs) from the genera *Acidovorax* and *Azoarcus* (Zhang et al., 2017), both of which were significantly enriched in SMBC treatments (Supplementary Figure S3).

The microbial reduction of As is primarily mediated by the arsenate reductase enzyme encoded by the *arsC* gene, which catalyzes the conversion of As(V) to As(III), and its abundance was positively correlated with the bioavailable As concentration. Across different water management conditions, arsenate reductase genes in all treatments exhibited an increasing trend, indicating active microbial involvement in As reduction. This reduction process typically occurs under aerobic conditions, although the *arsC* gene is expressed in both aerobic and anaerobic microorganisms (Silver and Phung, 2005). The *arsC* gene was well expressed during the incubation, while As(III) could hardly be detected during the dry period, indicating that the *arsC* gene was not well transcribed.

The abundance of As methylation-related genes, including *arsM*, increased as cultivation progressed, with no significant variations in gene abundance observed across treatments. The concentration of *arsM* genes in rice soil correlates with the levels of methylated As within the soil-rice system and is influenced by soil properties such as pH. Furthermore, anaerobic conditions enhance the abundance and activity of anaerobic As-methylating microorganisms that convert As(V) to As(III) under these conditions (Mlangeni et al., 2019). During the initial stages of cultivation, the concentrations of monomethylarsonic acid (MMAs) and dimethylarsinic acid (DMAs) remain low, and organic As is undetectable in later stages. This phenomenon may result from the high concentrations of As(III) and As(V), which are toxic to microorganisms during the soil cultivation process. This toxicity likely impairs the microbial capacity for As methylation. These findings align with the studies that highlight the adverse effects of elevated As concentrations on microbial communities, which consequently diminish the efficiency of As methylation in soils (Zhai et al., 2017). At the same time, some studies have shown that sulfate-reducing bacteria can also affect the methylation expression of As (Chen et al., 2020).

### 4.3 SMBC decreased the bioavailability of Cd and As in the soil

The addition of SMBC significantly reduces Cd bioavailability in soil by regulating Cd speciation through adsorption, pH regulation, and microbial transformations. SMBC immobilizes Cd by promoting electrostatic interactions between Cd^2+^ and negatively charged surfaces, effectively binding Cd^2+^ to soil particles. Compared to BC, SMBC achieves greater Cd reduction due to its higher surface area and more functional groups, which provide additional adsorption sites. SMBC also increases soil pH, resulting in decreased Cd bioavailability. By raising soil pH, silicon-containing materials lower hydrogen ion concentrations, creating negatively charged soil surfaces that attract and adsorb Cd^2+^ (Yang et al., 2019; Wang et al., 2016). Silicon in SMBC can also react with Cd to form insoluble silicate precipitates, further reducing Cd bioavailability and plant uptake. Additionally, microbial activity influences Cd bioavailability through the presence of Cd-related functional genes. The *DsrB* gene, associated with microbial Cd resistance, demonstrates a negative correlation with bioavailable Cd concentrations (*p* < 0.01).

SMBC upregulates *DsrB* gene expression via several mechanisms. Iron oxides present in SMBC serve as electron acceptors, enhancing *SRB* activity and thereby promoting *DsrB* expression. Additionally, silicon improvement creates favorable conditions for microbial activity, while changes in soil pH and DOC further support *SRB* activity (Ueki et al., 2022). A negative correlation between *DsrB* expression and bioavailable As suggests that lower As concentrations alleviate *SRB* suppression, thereby enhancing their activity and further upregulating *DsrB* gene expression.

In parallel, the bioavailability of As in soil is influenced by DOC, material complexation, and microbial activity. DOC competes with As for adsorption sites, thereby increasing the As concentration in the soil solution. SMBC application results in reduced DOC levels, mitigating this competition and lowering As bioavailability. SMBC promotes the formation of silica-metal oxide complexes with iron and aluminum, facilitating the co-precipitation of As. The presence of Ca^2+^ in SMBC further enhances As immobilization by forming stable complexes with As(V) (Herath et al., 2020; Gao et al., 2022). Microbial activity also modulates As speciation in the soil through oxidation, reduction, and methylation processes(Wang et al., 2022; Sonthiphand et al., 2024). SMBC treatments mainly enhance *aioA* expression by improving microbial habitats and enabling interactions between iron oxides and silicon in biochar. The *arsC* gene increases with SMBC addition, although its activity is suppressed when alternative pathways dominate (Escudero et al., 2013). Notably, the immobilization capacity for As is significantly greater in the SMBC2 and SMBC3 composites compared to SMBC1, resulting in a more effective reduction of As bioavailability. Taken together, the results suggest that the addition of 5% calcium silicate-magnetized biochar (5% SMBC2) is an efficient strategy for immobilizing bioavailable Cd and As in contaminated soils, thereby mitigating the potential risks associated with these toxic metals.

## 5 Conclusion

This study investigates the efficacy of silicon–iron-modified biochars (SMBCs) in regulating cadmium (Cd) and arsenic (As) speciation in Cd- and As-contaminated paddy soils. The results from soil incubation experiments indicate that SMBCs effectively modulate heavy metal speciation, enhance soil properties, and stimulate microbial activity, leading to a significant reduction in the bioavailability of Cd and As. Specifically, SMBC application increased soil pH levels and dissolved organic carbon (DOC) concentrations. By the end of the incubation, SMBC additives significantly elevated residual Cd concentrations by 2.94–16.17% (*p* < 0.05) and As(V) concentrations by 11.42–26.07% (*p* < 0.05). The addition of SMBCs enriched the abundance of *aioA* while also enhancing sulfate-reducing bacterial activity, which resulted in increased expression of the *DsrB* gene. Cd was transformed into a stable residual form through electrostatic adsorption, silicate precipitation, and sulfate reduction mediated by the *DsrB* gene.

Stabilization occurs through microbial oxidation to the less toxic and mobile form of As(V), driven by the *aioA* gene, and through complexation with iron, aluminum, and calcium, which promote the transfer of As to a stable state. Among the different SMBC additives tested, calcium silicate-magnetic biochar (SMBC2) exhibited the most significant efficacy, effectively reducing the bioavailability of Cd and As and transforming them into less toxic and less mobile forms. This study highlights the potential of calcium silicate-magnetic biochar as an effective additives for remediating Cd and As contamination in paddy soils. While the short-term efficacy of silicon–iron-modified biochars (SMBCs) in immobilizing Cd and As has been demonstrated, their long-term stability and performance require further exploration. Environmental factors, such as redox fluctuations, pH variations, and changes in soil organic matter or microbial activity, may dynamically influence metal-biochar interactions. Future research should prioritize field trials or controlled pot experiments to assess the sustained impacts of SMBCs on soil health, crop productivity, and metal(loid) bioavailability under diverse environmental conditions.

## Data Availability

The qPCR datasets presented in this study can be found in online repositories. The names of the repository/repositories and accession number(s) can be found in the article/Supplementary material. The rest data would be available by reasonable request from corresponding author.

## Footnotes

^1^ https://doi.org/10.6084/m9.figshare.28435226.v2
