# Supplementary material for: Silicon-iron modified biochar remediates cadmium and arsenic co-contaminated paddy soil by regulating cadmium and arsenic speciation
Source: Front Microbiol. 2025 Apr 11;16:1579213. doi: 10.3389/fmicb.2025.1579213 (PMC12041675; doi:10.3389/fmicb.2025.1579213)
Supplement: Supplementary file 1 [file Data_Sheet_1.DOCX]

**Slicon-iron modified biochar remediates cadmium and arsenic co-contaminated paddy soil by regulating cadmium and arsenic speciation**

Yao Chen^a,b^, Xin Tian^a,b^, Jia-hao Wang^a,b^, Yu Zhang^a,b^, Jie Wang^a,b^, Zhang-tao Li^c^, Ke-li Zhao^a,b^, Ji-zi Wu^a,b*^

*^a^ College of Environment and Resources, Zhejiang A&F University, Lin 'an 311300, China;*

*^b^ Zhejiang Provincial Key Laboratory of Soil Pollution Bioremediation, Zhejiang A&F University, Lin 'an 311300, China*

*^c^ Key Laboratory of Recycling and Eco-Treatment of Waste Biomass of Zhejiang Province, School of Environment and Natural Resources, Zhejiang University of Science and Technology, Hangzhou 310023, China*

^*^ *Corresponding author:* Ji-zi Wu (*[jiziwu@zafu.edu.cn](mailto:jiziwu@zafu.edu.cn))*

**Supporting Information**

This file contains 3 Figures.

1. The XPS spectra of As*3d* and Cd*3d* for silicon-iron modified biochar recovered from soil


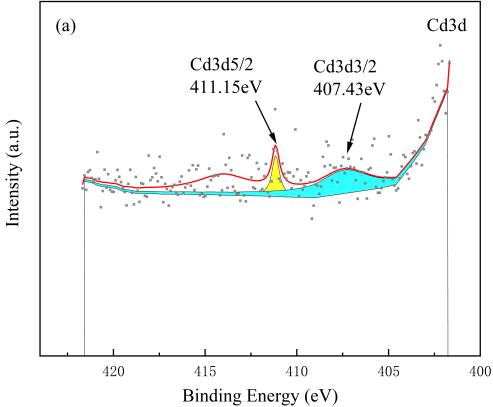

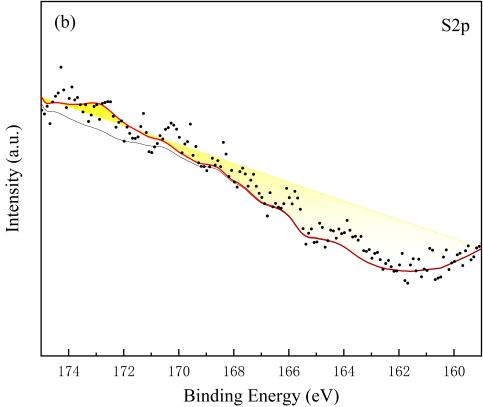


Figure. S1. The XPS spectrum of Cd*3d* (a) and S2*p* (b) in the soil (S and Cd shows almost no signal).


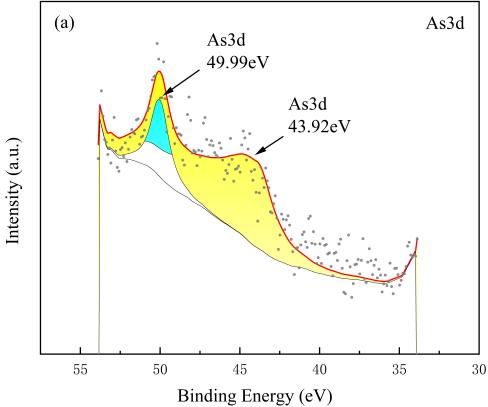

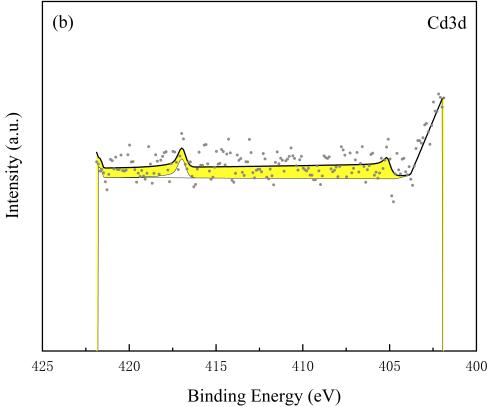


Figure. S2. The XPS spectra of As*3d* (a) and Cd*3d* (b) for silicon-iron modified biochar recovered from soil

1. The abundance changes of *Acidovorax* and *Azoarcus* in soil.


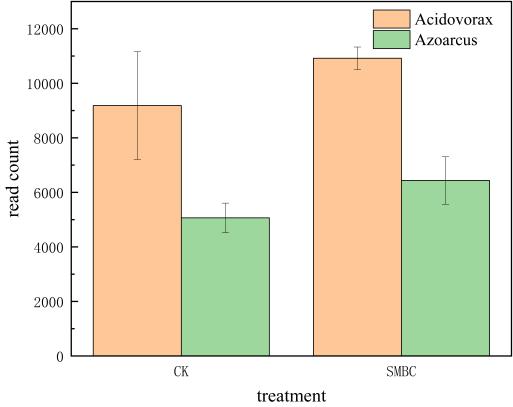


Figure. S3. The abundance changes of *Acidovorax* and *Azoarcus* in soil after the addition of materials.

Metagenomic Sequencing Method: The metagenomic sequencing was completed by Shanghai Personalbio Biotechnology Co., LTD. Total microbial genomic DNA samples were extracted using the OMEGA Mag-Bind Soil DNA Kit (M5635-02) (Omega Bio-Tek, Norcross, GA, USA), following the manufacturer’s instructions, and stored at -20°C prior to further assessment. The quantity and quality of extracted DNAs were measured using a Qubit™ 4 Fluorometer, with WiFi: Q33238 (Qubit™ Assay Tubes: Q32856; Qubit™ 1X dsDNA HS Assay Kit: Q33231) (Invitrogen, USA) and agarose gel electrophoresis, respectively. The extracted microbial DNA was processed to construct metagenome shotgun sequencing libraries with insert sizes of 400 bp by using Illumina TruSeq Nano DNA LT Library Preparation Kit. Each library was sequenced by Illumina NovaSeq platform (Illumina, USA) with PE150 strategy at Personal Biotechnology Co., Ltd. (Shanghai, China).
